# Multifunctional nanoplatforms co-delivering combinatorial dual-drug for eliminating cancer multidrug resistance

**DOI:** 10.7150/thno.59342

**Published:** 2021-04-19

**Authors:** Xiao Wei, Mingzhu Song, Weijie Li, Jing Huang, Guang Yang, Yi Wang

**Affiliations:** 1 School of Preclinical Medicine, Chengdu University, Chengdu 610106, P. R. China.; 2 Evidence-Based Medicine Center, West China Hospital, Sichuan University, Chengdu, 610041, P. R. China.; 3 College of Medicine, Southwest Jiaotong University, Chengdu 610031, P. R. China.; 4 School of Life Science and Engineering, Southwest Jiaotong University, Chengdu 610031, P. R. China.

**Keywords:** co-delivery, dual-drug, multifunctional nanoplatform, multidrug resistance, cancer therapy

## Abstract

Clinically, the primary cause of chemotherapy failure belongs to the occurrence of cancer multidrug resistance (MDR), which directly leads to the recurrence and metastasis of cancer along with high mortality. More and more attention has been paid to multifunctional nanoplatform-based dual-therapeutic combination to eliminate resistant cancers. In addition to helping both cargoes improve hydrophobicity and pharmacokinetic properties, increase bioavailability, release on demand and enhance therapeutic efficacy with low toxic effects, these smart co-delivery nanocarriers can even overcome drug resistance. Here, this review will not only present different types of co-delivery nanocarriers, but also summarize targeted and stimuli-responsive combination nanomedicines. Furthermore, we will focus on the recent progress in the co-delivery of dual-drug using such intelligent nanocarriers for surmounting cancer MDR. Whereas it remains to be seriously considered that there are some knotty issues in the fight against MDR of cancers via using co-delivery nanoplatforms, including limited intratumoral retention, the possible changes of combinatorial ratio under complex biological environments, drug release sequence from the nanocarriers, and subsequent free-drug resistance after detachment from the nanocarriers. It is hoped that, with the advantage of continuously developing nanomaterials, two personalized therapeutic agents in combination can be better exploited to achieve the goal of cooperatively combating cancer MDR, thus advancing the time to clinical transformation.

## Introduction

According to global cancer statistics, there have been 18.1 million new cases and 9.6 million cancer deaths worldwide 1. So, cancer is still one of the deadliest malignant diseases 2. Chemotherapy is the leading clinical treatment modality of cancers nowadays 3, but one long-term challenge to this conventional strategy is the occurrence of cancer multidrug resistance (MDR) 2, 4, which becomes the simultaneous development of resistance to structurally and mechanistically unrelated drugs 5. About half a million new cancer cases show the state of MDR every year while undergoing chemotherapy 6, which leads to a large number of cancer metastases and relapses, accounting for nearly 10% to 90% 7. Especially, it accounts for over 90% of chemotherapy failures in clinical metastatic cases 8, 9. Accordingly, higher doses of the chemotherapeutics usually need to be administrated with high frequency, bringing about serious toxicity that directly affects the survival time of cancer patients 10, 11.

**Figure 1:**
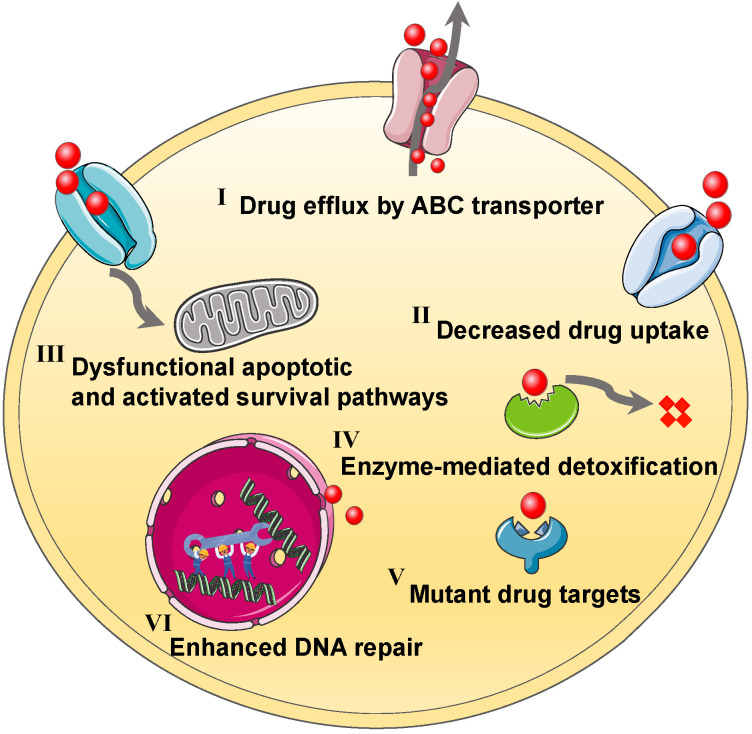
Schematic illustration of various MDR mechanisms, including (I) drug efflux by ABC transporter, (II) decreased drug uptake, (III) dysfunctional apoptotic and activated survival pathways, (IV) enzyme-mediated detoxification, (V) mutant drug targets, (VI) enhanced DNA repair. Abbreviations: MDR: multidrug resistance; ABC: ATP-binding cassette.

Tumor genetic diversity often gives rise to temporary responses to chemotherapy agents, followed by multiple drugs tolerance resulting from various complex mechanisms, including intrinsic resistance or acquired resistance in the cancer cells 12-14. In detail, the development of MDR may be classified into the following pathways (**Figure 1**): the changes of drug influx/efflux, the enhancement of DNA repair capacity, the alteration of drug metabolism that enables the detoxification, the mutations of drug targets, and the activation of parallel signal pathways 15, 16. It follows that the MDR mechanisms can often easily alter other pathways to allow the cancer cells to thrive, and ultimately resulting in ineffective chemotherapy 17. To circumvent the MDR, co-administration of two kinds of agents that can concurrently tackle various survival routes in tumor cells is powerful for addressing the MDR and reinforcing antitumor potency 18. Meanwhile, this combined strategy may regulate the genetic blocks responsible for cancer cell mutations and postpone the adaption process of drug resistance. Furthermore, while both drugs are adopted in combination for treating the resistant cancers, the ideal combination pattern with an optimized ratio can be selected out based on underlying MDR mechanisms. For instance, chemotherapeutic agents can be mixed with MDR reversal inhibitors (e.g., P-glycoprotein (P-gp) inhibitor), tyrosine kinase inhibitors or pro-apoptotic agents, to collaboratively conquer the MDR of cancers 19. Although various resistance suppressants or reversal reagents have been developed for clinical use in last decades, they still face the same problems as free small-molecule drugs, such as short half-life, severe toxic side effects, or poor therapeutic activity, thereby limiting their clinical efficacy 20, 21. Fortunately, the emerging of nanotechnology can serve to resolve these dilemmas by simultaneous delivering double payloads, increasing drug solubility, improving pharmacokinetic distribution, enhancing accumulation and retention, and controlling drug release on demand 4, 22. The relevant studies focusing on nanotechnology-based combination therapy have sharply increase within the past decades, thus more and more efforts have been devoted to the nanosized drug delivery systems co-delivering dual drugs for synergistic antitumor 4. In this context, a variety of multifunctional nanoplatforms can be designed for carrying the rational combination of dual cargoes based on the relevant resistance mechanisms, in order to achieve the optimal tumor inhibition effect by eliminating drug resistance in advance.

In this review, we first introduce various categories of co-delivery nanoplatforms and their functional diversities that contain specific cell targeting and stimulus-responsive release. Then we emphatically collate and summarize the detailed strategies for circumventing the MDR based on the co-delivery of two types of therapeutic agents by using nanocarriers. Last, we make an in-depth discussion on the applications of co-delivery nanoplatforms to overcome MDR mechanisms in cancer therapy, and summarize the existing challenges in the current therapeutic strategies and in clinical use. From this, we offer our own insights to improve the combinatorial therapy modalities based on multifunctional nanoplatforms, laying a theoretical foundation for future clinical trials. In addition, some nanoplatforms possessing the potential to clinically develop co-delivery nanomedicines are briefly prospected. Altogether, this review focuses on recent research publications and attempts to explore various therapeutic ways, in which advanced nanoplatforms can be designed to better deliver both drugs simultaneously, thus tenaciously combating cancer MDR from multiple possible angles.

## Rational design of multifunctional nanoplatforms for co-delivering dual drugs

Co-delivery of dual-drug based on nanoplatforms may become a powerful strategy that contributes to a perfect efficacy of anti-MDR. Generally, by virtue of the drug-carrying capacity of the nanocarriers, the dual-drug package with different physicochemical properties can be delivered simultaneously and maintain a certain combination ratio. Also, nanocarriers carrying combinatorial drugs can easily access the tumor site by enhanced permeability and retention (EPR) effect (a passive targeting mode) or ligand-mediated active targeting 23. Additionally, stimulus-responsive nanocarriers can control the release of two drugs under certain stimulating circumstances, thereby increasing treatment concentration. After entering the cells in endocytic manner, the dual-drug-loaded nanocarriers encapsulated into endo/lysosomes can help preferentially evade the MDR 24, and then responsively facilitate endosomal escape of both drugs into the cytoplasm to reverse the corresponding MDR pathway, thus recovering intracellular contents of therapeutants 25. Accordingly, rational design of multifunctional nanoplatforms, holding appropriate nano-structure/morphology, targeting capacity and stimulus response, will be extremely beneficial to co-deliver dual-drug for eliminating the MDR of cancers in a recent perspective 26.

### Different types of nanosystems for co-delivering dual-drug

**Figure 2:**
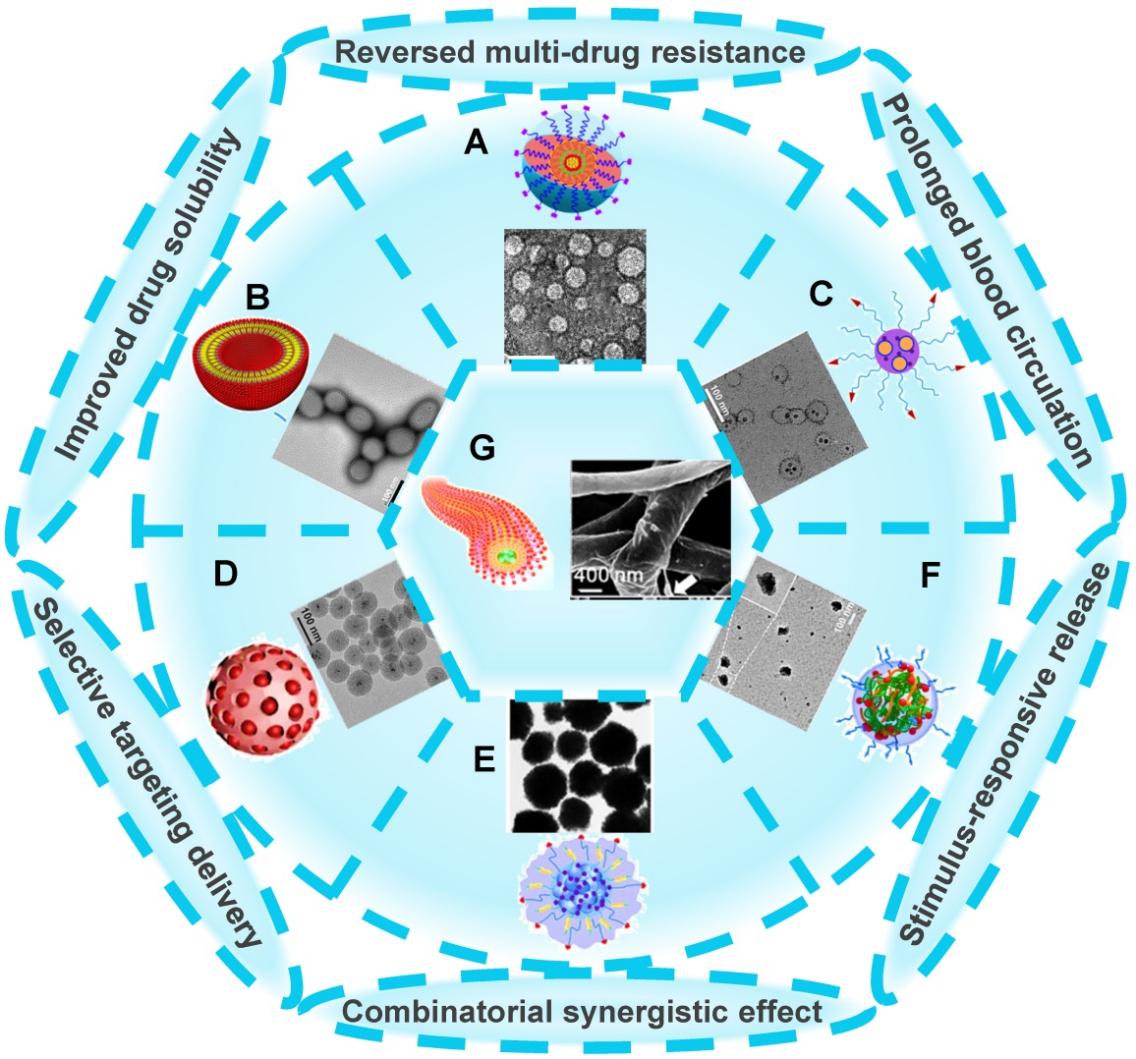
Various nanocarrier platforms for co-delivering dual therapeutics, including (A) polymeric micelle, reproduced with permission from Ref. 27, Copyright © 2016, Wiley-VCH Verlag GmbH & Co. KGaA, Weinheim. (B) Liposome, reproduced with permission from Ref. 28, Copyright © 2017, Wiley-VCH Verlag GmbH & Co. KGaA, Weinheim. (C) Polymeric nanoparticle, reproduced with permission from Ref. 29, Copyright © 2014, American Chemical Society. (D) MSN, reproduced with permission from Ref. 30, Copyright © 2020, American Chemical Society. (E) Fe_3_O_4_ nanoparticle, reproduced with permission from Ref. 31, Copyright © 2020, American Chemical Society. (F) Polymeric nanogel, reproduced with permission from Ref. 32, Copyright © 2018, American Chemical Society, and (G) nanofiber, reproduced with permission from Ref. 33, Copyright © 2019, American Chemical Society. Abbreviations: MSN: mesoporous silica nanoparticle; Fe_3_O_4_: iron oxide.

There are a wide variety of nanoscale drug delivery systems, including micelles, liposomes, polymeric nanoparticles, mesoporous silica nanoparticles (MSNs), etc. Herein, we mainly emphasize the design of the nanocarriers based on material category for developing ideal co-delivery nanotherapeutics (**Figure 2**), characterized with increased solubility, prolonged circulation, targeting delivery, on-demand release, reversed resistance and synergistic treatment. Additionally, a summary of co-delivery of dual-drug through different nanocarriers for treating various cancers is displayed in **Table 1**. It also reflects that different kinds of nanocarriers possess diverse drug-loading mechanisms due to their unique structural characteristics, which facilitate the co-encapsulation of two drugs with different physicochemical properties into one single nanosystem, and is further conductive to the synergistic treatment of cancers.

#### Polymeric micelles

Polymeric micelle with core-shell structure can be developed by the self-assembly of amphiphilic polymers in aqueous solution, which is often used as a drug delivery nanocarrier to improve the hydrophobicity and bioavailability of drugs. The micellar hydrophilic shell is usually modified with poly(ethylene glycol) (PEG), which can prolong the drug circulation in blood. If the hydrophilic moiety in micelles is composed of cationic polymer, which can bind negatively charged drugs (e.g., nucleic acid drugs) via electrostatic forces. Besides, their hydrophobic inner core can be easily loaded with lipid-soluble drugs through hydrophobic interaction. Accordingly, micelles can be served as a suitable vehicle for simultaneously co-loading two drug models with different physicochemical properties. Generally, micellar carriers are commonly designed for carrying two chemotherapeutic agents 34, 50, 51. For example, Lv and co-workers developed a micelle consisting of amphiphilic methoxy poly(ethylene glycol)-b-poly(L-glutamic acid)-b-poly(L-lysine) triblock copolymer decorated with deoxycholate (mPEG-b-PLG-b-PLL/DOCA), where the outer PEG segment extended the blood circulation, the middle PLG shell loaded the adriamycin (ADM) via electrostatic forces, and hydrophobic DOCA modified PLL encapsulated another chemotherapeutic paclitaxel (PTX) through hydrophobic interaction 35. The experimental outcomes demonstrated that co-delivery of ADM and PTX using micelles exhibited synergistic antitumor effect on the A549 lung cancer.

Otherwise, the micellar structure can be utilized as a vector for delivering the combined chemotherapeutic and gene-based agents (siRNAs or microRNAs) 36, 52. Chen et al. reported that a triblock copolymer composed of poly(2-(diisopropyl amino)ethyl methacrylate) (PDPA), poly(N-(2,2′-dithiobis(ethylamine)) aspartamide) PAsp (AED) and PEG, which formed a nanomicelle that could encapsulate doxorubicin (DOX) via hydrophobic force and anti-apoptotic Bcl-2 siRNA by electrostatic adsorption 53. As a result, the administration of Bcl-2 siRNA significantly aggravated DOX-mediated Bcl-2 down-regulation, leading to synergistically increased apoptosis of SKOV-3 ovarian cancer cells. Shi et al. also synthesized a type of triblock copolymer based on MPEG-PCL-g-PEI to prepare the cationic micelles, thereby effectively co-delivering chemotherapeutic drug and functional gene for enhancing tumor-suppression potency 37.

#### Polymeric nanoparticles

Polymer nanoparticles own a polymerized solid core, which can facilitate the loading of hydrophobic drugs by mixing the drugs with the polymer solution 54. Namely, while the polymers self-assemble into particles, drug molecules are physically trapped in nanoparticles via hydrophobic or electrostatic forces. This class of nanocarrier can be developed by the self-assembly of various biodegradable copolymers like poly(lactic-co-glycolic acid) (PLGA), polysaccharides or poly-L-lactide (PLA) 38, 55, 56, characterized with structural stability, uniform size distribution and programmed drug release. For PLGA nanoparticles, Guo et al. developed spherical PEGylated PLGA nanoparticles with a uniform size of approximately 50 nm, which physically co-encapsulated rapamycin (RAPA) and cisplatin (CDDP) for synergistic enhanced antitumor activity, alongside RAPA could sensitize A375 melanoma cells to CDDP 57. Also, Rammohan et al. synthesized PEGylated PLGA polymeric carrier loading with two antisense-miRNAs through electrostatic bonding, simultaneously combating the anti-apoptosis and metastasis induced by miRNAs 39. For polysaccharide-based nanoparticles, chondroitin sulfate (CS), dextran (DEX), hyaluronic acid (HA) and chitosan have often been used to form polymeric nanoparticles. It has been reported that a protoporphyrin (PpIX)-conjugated CS can self-assemble into nanoparticles, which concurrently loaded a resistance reversal agent Apatinib (APA) and an anticancer drug DOX by hydrophobic forces, and successfully reversed the MDR in breast cancer via Apa-improved DOX sensitivity 55. Wang et al. fabricated polymeric nanoparticles composed of DEX 58, which simultaneously encapsulated PpIX via covalent grafting and the anticancer drug camptothecin (CPT) via hydrophobic interaction for chemo-photodynamic therapy. Deng et al. designed a novel nanoparticle based on HA and chitosan by iontropic gelation technique, then incorporating MiR-34a mimic and DOX via electrostatic interaction 59. For PLA nanoparticles, the form of PLA-drug conjugates is synthesized by adapting metal alkoxide chemistry, and then these polymer-drug prodrugs self-assemble into polymeric nanoparticles through a single-step nanoprecipitation method. Namely, drug compounds are incorporated into single polymeric nanoparticles through covalent conjugation, resulting in a high loading efficiency. Aryal et al. used DOX and CPT as two chemotherapeutic drugs to separately develop DOX-PLA and CPT-PLA prodrugs, then loading into the lipid-coated polymeric nanoparticles with over 90% loading efficiency 56.

#### Liposomes

Liposomes usually consists of phosphatidylcholine, phosphatidylethanolamine and cholesterol, presenting as spherical vesicle with a lipid bilayer shell and an inner aqueous core, which can enable the concurrent co-delivery of lipophilic and hydrophilic drugs 60. Drug encapsulation into liposomes is achieved by either active extrusion or passive diffusion through the lipid bilayers. Specifically, three types of drugs present different loading ways of liposomes, including lipophilic drugs locating in the lipid bilayer shell via hydrophobic interaction, hydrophilic drugs locating in aqueous inner core via passive diffusion, and nucleic acid drugs locating in the cationic liposomal surface via electrostatic adsorption. Based on these loading properties, liposome-mediated co-delivery of combinatorial dual-drug becomes imperative and worthy of development. Generally, liposomes are often used to carry two chemotherapeutic drugs or a combination of chemotherapeutic and nucleic acid drugs 61, 62. For example, Zhang et al. investigated a method that 1,2-Distearoyl-sn-glycero-3-phosphoethanolamine-N-methoxy(polyethyleneglycol) (DSPE-PEG2000), dipalmitoyl-sn-glycero-3-phosphocholine (DPPC), soybean phosphatidylcholine (SPC) and cholesterol (CHOL) were used to prepare liposomes, for co-encapsulating dexamethasone (DAT) and docetaxel (DTX) into hydrophobic lipid bilayer shell and achieving the property of continuous drug release 63. In another research, liposomes were constructed by the self-assembly of DSPE-PEG2000, soybean lecithin (S100) and CHOL (3:1:1, w:w:w) 42, to co-deliver a combination of tumor necrosis factor-related apoptosis-inducing ligand (TRAIL) and PTX for enhanced anti-melanoma effects. Of which, TRAIL was attached to negatively charged liposomal surface via electrostatic adsorption while PTX was physically encapsulated inside the lipid bilayer shell. In addition, liposomal structure can also be used to co-deliver chemotherapeutics and nucleic acid drugs (e.g., siRNA). To this end, in addition to encapsulating chemotherapeutic drugs into the lipid bilayer or aqueous inner core, the introduction of cationic 1,2-Dioleoyl-3-Trimethylammonium-Propane (DOTAP) or the positive protamine into liposomes will be beneficial to compress and load nucleic acid drugs through electrostatic forces 43, 62, 64.

#### Other nanocarriers

Other types of nanomedicines have also been studied for synchronously co-delivering dual payloads, such as MSNs 46, 65, iron oxide (Fe_3_O_4_) nanoparticles 66, nanofibers 33, 67, nanogels 32, 68 and carbon nanomaterials 49, 69, etc. Among them, MSNs are an effective and easily functionalized vehicle for controlled drug release through stimulus-responsiveness. For example, Li et al. developed a stimulus-responsive nanocarriers based on MSNs for co-entrapping CPT and DOX, resulting in the synergistic chemotherapy against the tumor 45. Additionally, for Fe_3_O_4_ materials-based nanoplatforms, Dutta et al. prepared surfactant-stabilized Fe_3_O_4_ magnetic nanocarriers through self-assembly of anionic surfactant and sodium dodecyl sulphate on hydrophobic (oleic acid coated) nanoparticles, which highly possessed water dispersibility and an average diameter of 10 nm, which were further applied to co-deliver DOX and curcumin (CUR) 66. For the research on nanogel-based nanocarriers, Wu et al. reported that the stimulus-responsive polymeric nanogels with less than 100 nm composed of poly(acrylic acid) were employed as co-delivery system for DOX and CDDP, to overcome the MDR of MCF-7/ADR human breast cancer cells 48. Furthermore, for carbon materials-based nanocarriers, Zhi et al. constructed this type of nanoplatforms based on PEI-modified graphene oxide (NGO) and polystyrene sulfonate, enabling co-delivery of anti-miR-21 and ADM for the reversal of MDR in MCF-7/ADR tumor cells 49.

### Various functionalities of nanocarriers for co-delivering dual-drug

**Figure 3:**
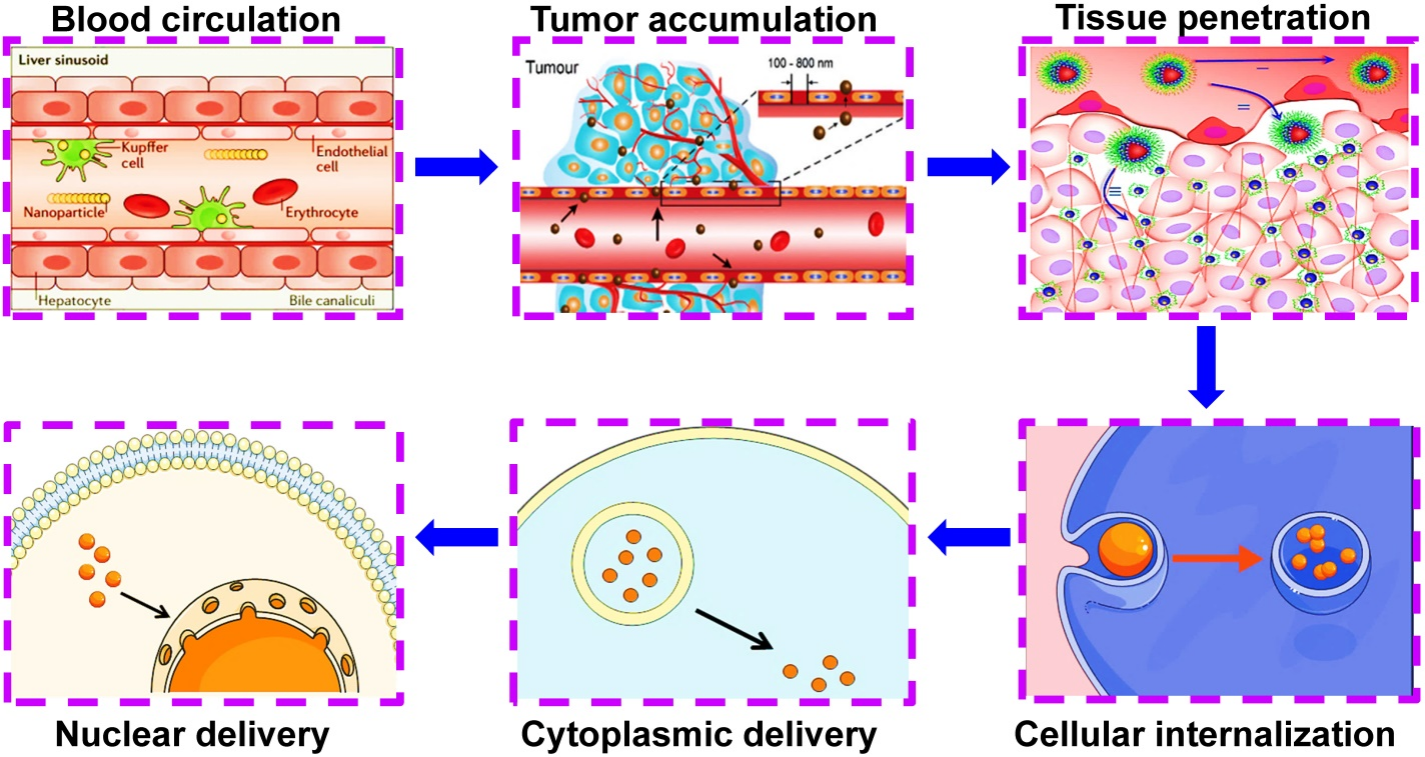
Nanocarriers can help transport therapeutic agents across a series of physiological barriers, thus successfully escaping from the reticuloendothelial system during blood circulation, completing EPR-mediated tumor accumulation, tumor microenvironmental delivery (the penetration of stromal barriers) and intracellular delivery (lysosomal escape, cytoplasmic release or organelle release). Reproduced with permission from Ref. 72, Copyright © 2017, Royal Society of Chemistry. Abbreviations: EPR: enhanced permeability and retention.

Ideal co-delivery nanocarriers should possess specific selectivity toward cancer cells, accompanied by triggered drug release in response to external stimuli (heat, light, ultrasound and magnetic field) or internal stimuli (pH, reduction, and enzyme) 70. By means of targeted and stimulus-responsive capacities, nanocarriers can help transport therapeutic agents across a series of physiological barriers, thus successfully completing tumor microenvironmental delivery (the penetration of stromal barriers) and intracellular delivery (lysosomal escape, cytoplasmic release or organelle release) (**Figure 3**) 71, 72. Herein, we will make a brief review on the targeted and stimulus-responsive co-delivery nanotherapeutics in cancer treatment.

#### Targeted nanocarriers

Targeted drug delivery with nanocarriers is crucial to improve treatment efficacy and reduce side effects. Generally, targeting is commonly divided into passive targeting mode (such as EPR effect) or ligand-mediated active targeting mode 73, 74. In this review, we will mainly emphasize the active targeting capacity of co-delivery nanocarriers endowed by a series of ligands. Among them, folate (FA) receptor is a desired drug delivery target due to its overexpression in most cancer cells 74, and FA-modified nanoplatforms have been investigated for the targeted co-delivery of dual-drug combination 48, 62. Also, transferrin (TF) is an ideal ligand that selectively binds to the transferrin receptor (TFR) overexpressed on the membrane of cancer cells 75, showing potential application in actively targeted drug delivery. Lang et al. employed TFR1-targeting liposomes to co-transport dual therapeutics to pancreatic tumor cells, facilitating efficient uptake by tumor cells with high expression of TFR1 44. Besides, some special peptides, regarded as targeting ligands, will endow the modified nanocarriers with an active targeting function 42. In a study by Li et al. 36, plectin-1 targeted peptide (PTP, NH_2_-KTLLPTP-COOH) was grafted on the dendrimer micelles for targeted co-delivery of nuclear receptor siRNA (siTR3) and PTX in pancreatic cancer treatment. Consequently, these PTP-modified micelles specifically accumulated in cancer cells through PTP-mediated cellular membrane-targeting. For another example, the integrin Rvβ3-specific ligand (RGD4C) and the cell-penetrating peptide TAT conjugated the poly(ethylene oxide)-block-poly(ε-caprolactone) (PEO-b-PCL) conferred the membrane penetration and active targeting to the micellar system, which facilitated specifically targeted co-delivery of siRNA and DOX to overcome MDR of breast cancer 76. Additionally, phagocytosis of nanotherapeutics can also be facilitated by the affinity between polysaccharides (e.g., HA or CS) and cell membrane receptors. Hence, these polysaccharide components have been used for targeted functionalization of nanocarriers, thus leading to specifically co-delivering dual drugs mediated by CD44 receptors overexpressed in many cancer cells 32, 55, 77.

#### Stimulus-responsive nanocarriers

Great efforts have been devoted to the exploration of responsive nanomedicines that respond to environmental stimuli. In this part, we will focus on the nanocarriers with different stimuli responses for controlled release of combined dual-drug.

Among many stimulants, pH sensitivity is most exploited to trigger drug release 78. Usually, pH 6.8-7.2 of tumor microenvironment and pH 5-6 of endosomes and pH 4.5-5.5 of lysosomes, as acid pH gradients, may be applied for responsive drug release in co-delivered nanocarriers 79, 80. According to the previous studies, co-delivery nanocarriers with a pH-response can respond to lysosomal acid stimulation through the modification of acid-labile groups (e.g., hydrazone bond) 45, 65, 81 or pH-sensitive polymers (e.g., PDPA) and poly(L-histidine) (PHis)) 53, 77, 82, thus facilitating the release of dual therapeutic payloads to effectively reverse tumor MDR. Besides, 2,3-dimethylmaleic anhydride (DMA), as an acid-sensitive compound, has been used to grafted onto diverse polymers like PLL 51, which made copolymer micelles easily change from a negative charge to a positive charge under the weak acid environment of tumor, which further benefited to enter the cancer cells and successfully released both drugs for the reversal of MDR. More importantly, pH-sensitive nanotherapeutics can rapidly release drugs inside the cells, thereby exceeding MDR-mediated drug efflux concentrations and achieving effective therapeutic concentrations that inhibit drug-resistant tumor cells 83.

The appearance of redox potential gradient in the microenvironment inside and outside tumor cells may be used as a stimulant to improve drug delivery efficiency 84. Among most reductive substances, glutathione (GSH) is a most used redox irritant. This is because GSH concentrations in tumor cytoplasm (~10 mM) is approximately 7 times higher than those in normal cells, and even higher in drug-resistant cancer cells 85. It has been demonstrated that disulfide bonds (-ss-), as covalently linked group, are usually used to respond to intracellular GSH-mediated redox stimulation, followed by thiol-disulfide exchange reactions 86. Accordingly, nano-drug delivery systems (e.g., MSNs and micelles) often introduce disulfide bonds to facilitate reductive responsive drug release, which may rapidly unload both therapeutic payloads upon exposure to GSH-rich tumor cytoplasm through self-disassembly, so as to maximize their anticancer potency 53, 65, 87. Furthermore, co-delivery of dual-drug by using redox-responsive nanocarriers may be in favor to overcome tumor MDR. For instance, Yin et al. designed a bio-reducible polymeric nanoparticle decorated with disulfide bonds for co-delivering combinatorial nucleic acid drugs, leading to a synergistic inhibition effect on the resistant tumor 88. Additionally, Sun's team reported the development of redox-responsive nanoplatforms, which was rapidly degraded with complete release of PTX and APA under the cellular enriched GSH, and resulted in effective circumvention of MDR in breast cancer 89.

Enzyme, as a common type of response stimulant, may be used to control drug release while enzyme-responsive nanomedicines locate in an enzyme-rich lysosomal environment or tumor stromal environment. Enzyme-sensitive moieties, such as esterase-sensitive ester linkages 90, 91 or matrix metalloproteinases-2 (MMP2)-responsive peptide segments 92, 93, are generally covalently incorporated into the nanosystems for triggered drug release under the tumor physical environment. For instance, Liang et al. fabricated a nanocapsule with a covalent modification of a hydrolyzable ester bond, facilitating quick release of combinatorial chemo-drugs through the hydrolysis of the ester bond mediated by the esterase 28. Interestingly, some metabolic or detoxification enzymes in MDR tumor cells are closely connected with the MDR mechanisms, which may be served as potential stimuli for controlled drug delivery in MDR cancer treatment 15. Whereas little attention has been paid to the exploitation of enzyme-responsive co-delivery nanocarriers for reversing the MDR of cancers.

Applications in other physical stimuli (e.g., magnetic field or ultrasound) have also been extensively explored for cancer combination therapy. Among these stimuli, the magnetic field-responsive nano-drug delivery system is closely related to the utilization of the magnetic materials 94. Lu et al. designed magnetic field-responsive Fe_3_O_4_-loaded MSNs for cancer thermo-chemotherapy, resulting in the quick heating and remotely triggered drug release upon exposure to a magnetic field 95. For the study on ultrasound as a stimulus, it has been generally reported that ultrasound-responsive nanoplatforms are usually designed for cancer chemo-sonodynamic therapy 96. Also, ultrasound-sensitive co-delivery nanosystems may be investigated for overcoming tumor MDR. In a study by Yin et al. 97, ultrasound-sensitive nanobubbles were formed by the hetero-assembly of micelles and liposomes, which were further used to simultaneously co-deliver PTX and siRNA for combating chemotherapeutic resistant hepatocellular carcinoma. Else, Baghbani et al. have investigated an ultrasound-responsive alginate/perfluorohexane nanodroplet for co-delivering DOX and CUR, which rapidly released dual payloads by low frequency ultrasound and exerted the reversal of MDR in ovarian cancer by the synergistic effects of both drugs 98.

## Overcoming drug resistance by co-delivery of combinatorial drugs using nanocarriers

Diverse types and functions in co-delivery nanoplatforms have been briefly described above. Taken together, whether these multifunctional nanocarriers can successfully deliver the two drugs into tumor cells is crucial for the subsequent reversal of drug resistance. Indeed, in addition to endow drugs good solubility, long metabolism, selective targeting and controlled release in time and space, these co-delivery nanocarriers may even reverse drug resistance through endocytic internalization. However, once the drug is released into the cytoplasm and converted into a free state, it is still subject to the effects of MDR mechanisms. To address it, the drug delivery systems should not only be rationally designed with multiple functions, but also deliver an optimized two-drug combination with the ability to evade or inhibit the MDR. Among various underlying MDR mechanisms, the altered expression of Bcl-2-related apoptotic proteins and the overexpression of adenosine triphosphate (ATP)-binding cassette (ABC) transporters severally represent intrinsic resistance and acquired resistance 99, 100. The former is independent of drug efflux pump, mainly related to mitochondrial apoptotic pathway involving the regulation of pro-apoptotic p53 transcriptional targets like Bax and anti-apoptotic factors like Bcl-2 101. The latter is closely associated with the upregulated expression of a family of energy-dependent drug efflux pumps (ABC transporters) 46, leading to the efflux of a variety of hydrophobic anticancer drugs such as DOX, CPT, and PTX. One of the most common transporter is P-gp (also known as MDR1), primarily existing in leukemia, liver, ovarian, breast, pancreatic cancers, etc. 101, 102. According to multiple target genes involved in different MDR mechanisms, we can freely choose the appropriate therapeutants that may modulate the corresponding MDR pathway to be combined with chemotherapy drugs, and then co-encapsulated in “two in one” nanosystems to increase chemosensitivity of cancer cells by reversing resistance. So far, a broad class of chemo-sensitizers have been developed to normalize the apoptosis pathway or suppress ABC drug transporters, such as ABC transporter inhibitors or pro-apoptotic agents 54.

Next, there is a main discussion about the studies of various combinatorial nanomedicines in overcoming different tumor MDR mechanisms. Herein, the MDR mechanisms of tumor are primarily involved in ABC transporter (P-gp)-mediated drug efflux and the aberrant expression of apoptosis-related protein. Of which, the descriptions of the reversal of ABC transporter-based MDR are showed in Section 3.1 and 3.2, and the discussions about the reversal of dysfunctional apoptosis-induced MDR are mainly presented in Section 3.3 and 3.4. Meanwhile, a research on overcoming abnormal DNA repair-mediated MDR is also described in the discussion of Section 3.4. It's worth noting that, there have been relatively few reports on co-delivery nanomedicines to overcome other resistance mechanisms like aberrant DNA repair, enzyme-mediated detoxification, and mutant drug targets. Thus we do not have a systematic summary of the researches on these related resistance mechanisms in this review. Instead, more efforts should be devoted to these studies. Additionally, different combinations of dual-drug in co-delivery nanocarriers for the reversal of MDR are summarized in **Table 2**.

### Co-delivery of two chemotherapeutic drugs

Chemotherapy as a first-line model in clinical cancer treatment is still highly favored. Generally, the combination of both chemical cytotoxins with different pharmacological properties can synergistically increase cytotoxicity in clinical trials. However, most chemotherapeutic drugs are identified as the substrates of the MDR, thereby limiting their clinical use 116. To solve this issue, the emerging of nanotechnology may not only help free chemical cytotoxins improve hydrophobicity, metabolism, bioavailability and targeting, but also circumvent MDR through lysosomal delivery. Nonetheless, there is still the possibility of pumping drugs out once the drug is delivered to the cytoplasm. Hence, co-delivery nanocarriers should be pluripotently designed and take full advantage of the merits of both chemical drugs that collaboratively overcome unfavorable drug resistance.

**Figure 4:**
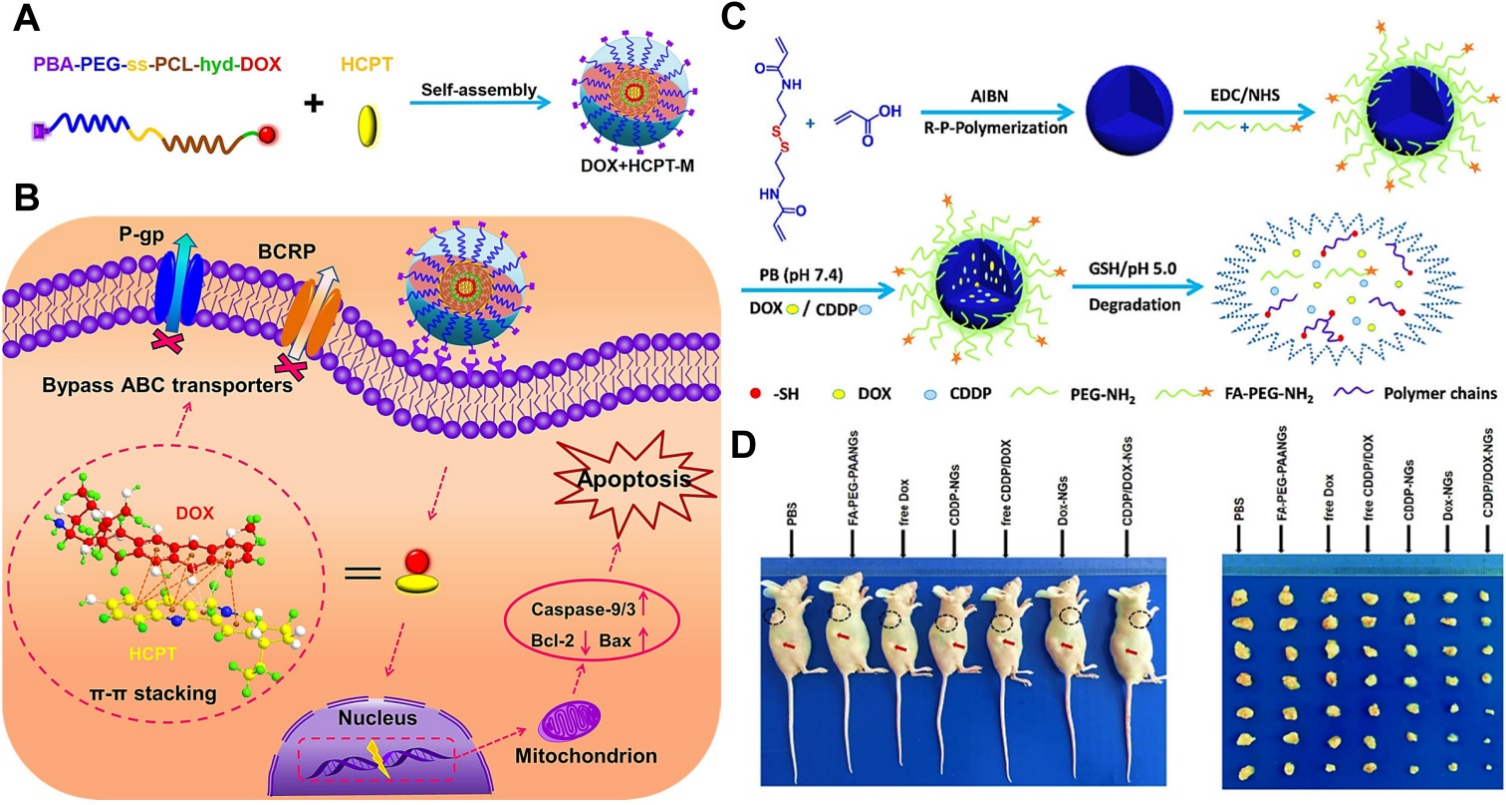
(A) The formation of DOX+HCPT-M micelles via the self-assembly of DOX-conjugated polymer with free HCPT. (B) Schematic illustration of co-delivery of a π-π stacked combination of DOX and HCPT using an intelligent micellar platform for overcoming breast cancer MDR. Reproduced with permission from Ref. 27, Copyright © 2016, Wiley-VCH Verlag GmbH & Co. KGaA, Weinheim. (C) Schematic illustration of the preparation, surface modification, dual-drug loading and drug releasing of the designed polymeric nanogels. (D) *In vivo* antitumor activity evaluation of different therapeutic formulations in BALB/c nude mice bearing MCF-7/ADR tumors. Reproduced with permission from Ref. 48, Copyright © 2017, American Chemical Society. Abbreviations: P-gp: P-glycoprotein; BCRP: breast cancer resistance protein; DOX: doxorubicin; HCPT: 10-hydroxycamptothecin; CDDP: cisplatin; FA: folate.

In our previous work, we developed targeted/pH/reduction-sensitive polymer micelles to co-deliver the combination of DOX and 10-hydroxycamptothecin (HCPT), for defeating the MDR in MCF-7/ADR breast cancer therapy (**Figure 4A-B**) 27. Since both traditional drugs have π-π conjugated aromatic moieties, they could be synchronously encapsulated to this single micellar platform through π-π stacking forces. As a result, the two chemotherapeutics released from these smart nanoplatforms could successfully bypass the recognition of drug efflux pumps (P-gp and BCRP) through a subtle change of molecular structure (**Figure 4B**), resulting from non-covalent π-π conjugation and collateral sensitivity 117. Yu et al. also reported that the combined DOX and HCPT doped in PLGA nanoparticles could indeed overcome MDR of cancer cells, thereby achieving enhanced therapeutic efficacy through synergistic effects between two chemotherapeutics 118. Besides, Wu and his colleagues investigated that DOX and CDDP were simultaneously incorporated into polymeric nanogels via π-π stacking, chelation and electrostatic force (**Figure 4C**) 48. Because this combined dual-drug formulation can not only overwhelm the cellular repair mechanisms 119, but also show obviously synergistic effect in phase III clinical trials 120, 121. Eventually, this co-delivery nanosystem could effectively overcome MDR in MCF-7/ADR cancer cells and exhibit significant antitumor efficacy (**Figure 4D**).

**Figure 5:**
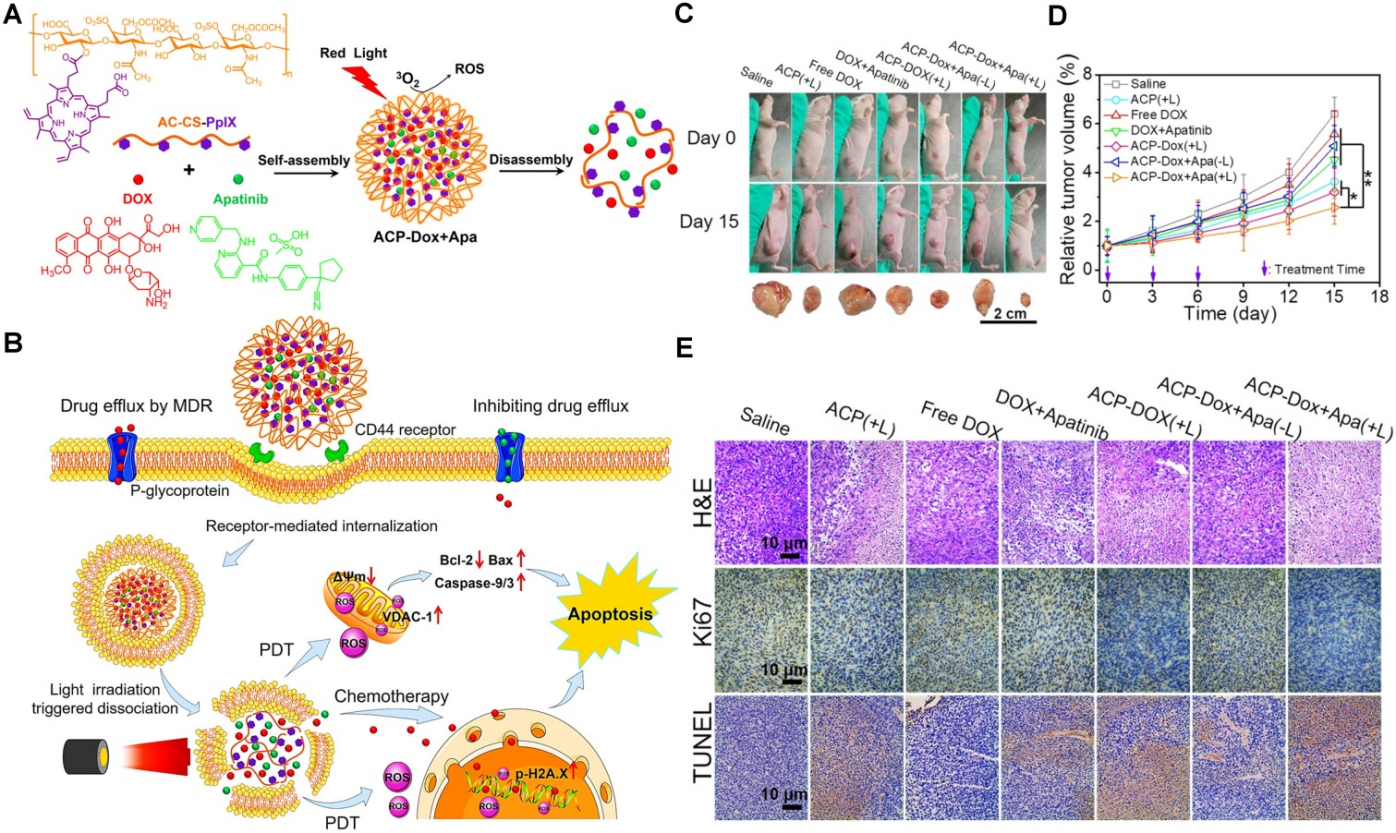
(A) The formation and light-triggered decomposition of versatile polymeric nanoparticles co-delivering anticancer therapeutic DOX and APA. (B) Schematic illustration of the mechanism of the reversal of cancer MDR via ACP-Dox+Apa nanoparticles. (C) The tumor tissue images of the MCF-7/ADR tumor-bearing nude mice. (D) Tumor growth profiles. (E) H&E, Ki67 and TUNEL analyses of tumor tissues from MCF-7/ADR xenografts-bearing nude mice. Reproduced with permission from Ref. 55, Copyright © 2018, American Chemical Society. Abbreviations: APA: apatinib; PpIX: protoporphyrin IX; ROS: reactive oxygen; PDT: photodynamic therapy; H&E: hematoxylin and eosin; TUNEL: terminal deoxynucleotidyl transferase-mediated dUTP nick-end labeling.

It has been reported that some tyrosine kinase inhibitors (TKIs) may either restore the apoptotic signaling pathway to sensitize resistant tumor cells to chemotherapeutics 122, or immediately attenuate the ATPase activity in P-gp to reinforce the cytotoxicity efficacy of antitumor 123. Therefore, co-delivery nanocarriers may be also constructed to combine TKIs with traditional chemical drugs, leading to regulating the cell survival/death-related mechanisms for overwhelming the MDR of cancers 124. For instance, APA, as one of TKIs, may observably waken P-gp-mediated MDR and potentiate the sensitivity of tumor cells to chemotherapeutic drugs 125. In our previous study, we designed a light-responsive polysaccharide nanoparticle loading with the combination of DOX and APA (**Figure 5A**) 55. When exposed to light irradiation, this nanoparticle instantly disassociated to release dual cargoes. After that, the released APA could recover the sensitivity of DOX by competitively inhibiting the P-gp activity, thus enhancing anticancer potency (**Figure 5B-E**). In addition, lapatinib (LPA), another type of TKI, may also reverse MDR of tumor cells by suppressing the P-gp. In a study by Wang et al., the micelles co-loading with DOX and LPA were developed for combating the resistant breast cancer, which successfully overcame the MDR through LPA-mediated inhibition of efflux pumps, and significantly promoting the *in vitro/in vivo* antitumor efficacy 103.

### Co-delivery of chemotherapeutic drugs and ABC transporter inhibitors

Macromolecular nanocarriers can directly evade the recognition of ABC transporters through endocytic pathways, contributing to taking the carried payloads away from the transmembrane multidrug efflux pumps 126. Afterwards, the nanocarriers release the dual drugs from lysosomes to cytoplasm, where both of them may still be expelled through the drug efflux pumps. Therefore, drug cargoes carried by nanocarriers should hold the ability to overwhelm the drug efflux kinetics. To this end, we need to choose one ideal agent for effectively relieving the resistance of cancer cells to the other chemotherapy drug. Here, we will focus on many kinds of drug-resistance modulators that can obviously reverse the MDR of cancers. First, several surfactants like _D_-α-tocopheryl polyethylene glycol 1000 succinate (TPGS) and pluronic P123, can be used to decrease the P-gp activity accompanied by the decline of ATP 77, 127. Second, siRNAs can be utilized to downregulate the expression of MDR-related genes for enhancing the sensitivity of resistant tumor cells to chemotherapeutics 128. Third, some chemosensitizers like verapamil can be employed for blocking ABC transporters. Last, some gaseous signaling molecules, such as nitric oxide (NO), hydrogen (H_2_) and sulfur dioxide (SO_2_), can be used as ABC transporter inhibitors to reverse the MDR by downregulating the expression level of P-gp.

#### Chemotherapeutic drugs and surfactants

**Figure 6:**
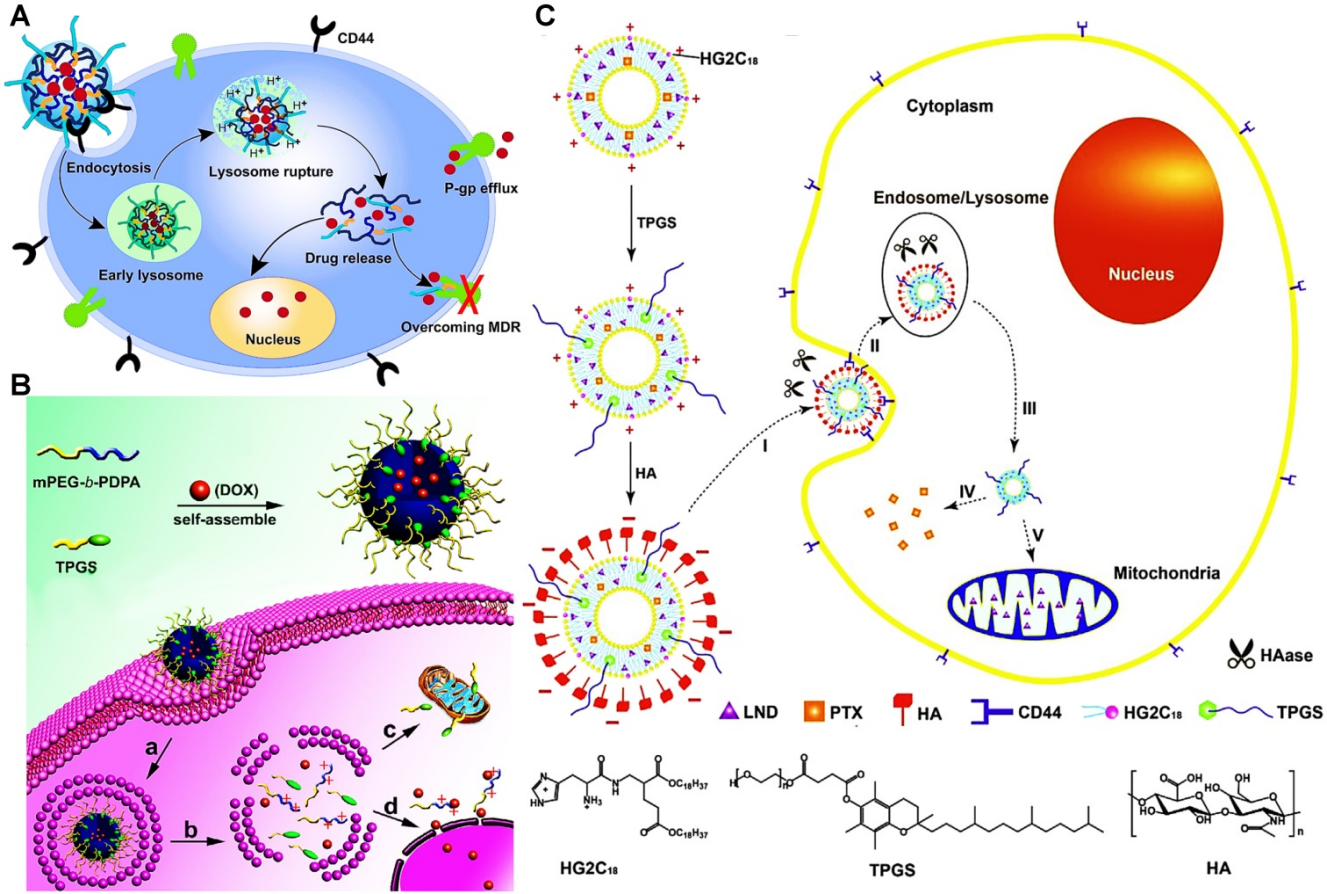
(A) Disclosure of the reversal mechanism of MDR via DOX-loaded micelles (HPHM/TPGS2000). After HPHM/TPGS2000 were selectively uptook by MCF-7/ADR cells via CD44 receptor-mediated endocytosis, pH-triggered release of TPGS2000 could suppress P-gp efflux pump to restore the chemosensitivity of DOX. Reproduced with permission from Ref. 77, Copyright © 2014, Elsevier. (B) The preparation and mechanism of mitochondria-targeted pH-responsive PDPA/TPGS@DOX micelles to overcome DOX resistance in breast cancer cells. Reproduced with permission from Ref. 131, Copyright © 2015, Elsevier. (C) Schematic design of multifunctional liposomes for co-delivery of TPGS and chemotherapeutics to reverse MDR in cancer treatment. Reproduced with permission from Ref. 133, Copyright © 2015, Elsevier. Abbreviations: TPGS: D-α-tocopheryl polyethylene glycol 1000 succinate; HA: hyaluronic acid; HG2C_18_: 1,5-dioctadecyl-N-histidyl-_L_-glutamate; LND: lonidamine; PTX: paclitaxel.

A broad range of P-gp inhibitors has been explored for bypassing P-gp-mediated MDR mechanism by blocking drug efflux. Among them, TPGS and pluronics are capable of playing a role in defeating the P-gp-mediated MDR through the inhibition of P-gp activity, and these polymeric inhibitors have good biosafety 129, 130. For example, Qiu et al. fabricated a mixed polymeric micellar system to co-deliver DOX and TPGS for combating MDR of breast cancer (**Figure 6A**) 77. By virtue of TPGS, a high amount of cellular uptake of dual-drug-loaded micelles was obtained in drug-resistant MCF-7/ADR tumor cells, attributed to the potentiated reversal effect of P-gp-induced MDR by TPGS, and restoring the sensitivity of anticancer DOX. Consequently, the micellar system exhibited higher and comparable cytotoxicity against MCF-7 cells and MCF-7/ADR cells, respectively. Meanwhile, it has been found that the micelles carrying TPGS can effectively suppress P-gp activity, resulting from the decline of mitochondrial membrane potential (MMP) and ATP, but without inhibition of P-gp expression. In another study, Yu et al. doped TPGS into the DOX-loaded micelles. As expected, TPGS successfully inhibited the P-gp-mediated MDR and restore the chemosensitivity of DOX-resistant MCF-7/ADR cells (**Figure 6B**) 131. In addition to using micelles as nanocarriers for co-delivering chemotherapeutics and TPGS, some studies have also reported that the development of PLGA nanoparticles or liposomes have been used to combine chemotherapeutic drugs with TPGS 132, 133, and finally indicating that these types of combinatorial nanomedicines have significant potential for efficiently combating drug resistance in cancer treatment (**Figure 6C**). In another work, He et al. introduced a surfactant cetyl trimethyl ammonium bromide (CTAB) identified as a structure-directing agent for developing MSNs loading with chemotherapeutic DOX, thus promoting the inhibition efficacy of ATP by CTAB, leading to enhanced reversal effect of P-gp efflux pump 113, 134. Consequently, the release of DOX from MSNs could continuously accumulate inside the MCF-7/ADR cells, and then induce the extensive apoptosis of MCF-7/ADR cells.

#### Chemotherapeutic drugs and siRNAs

**Figure 7:**
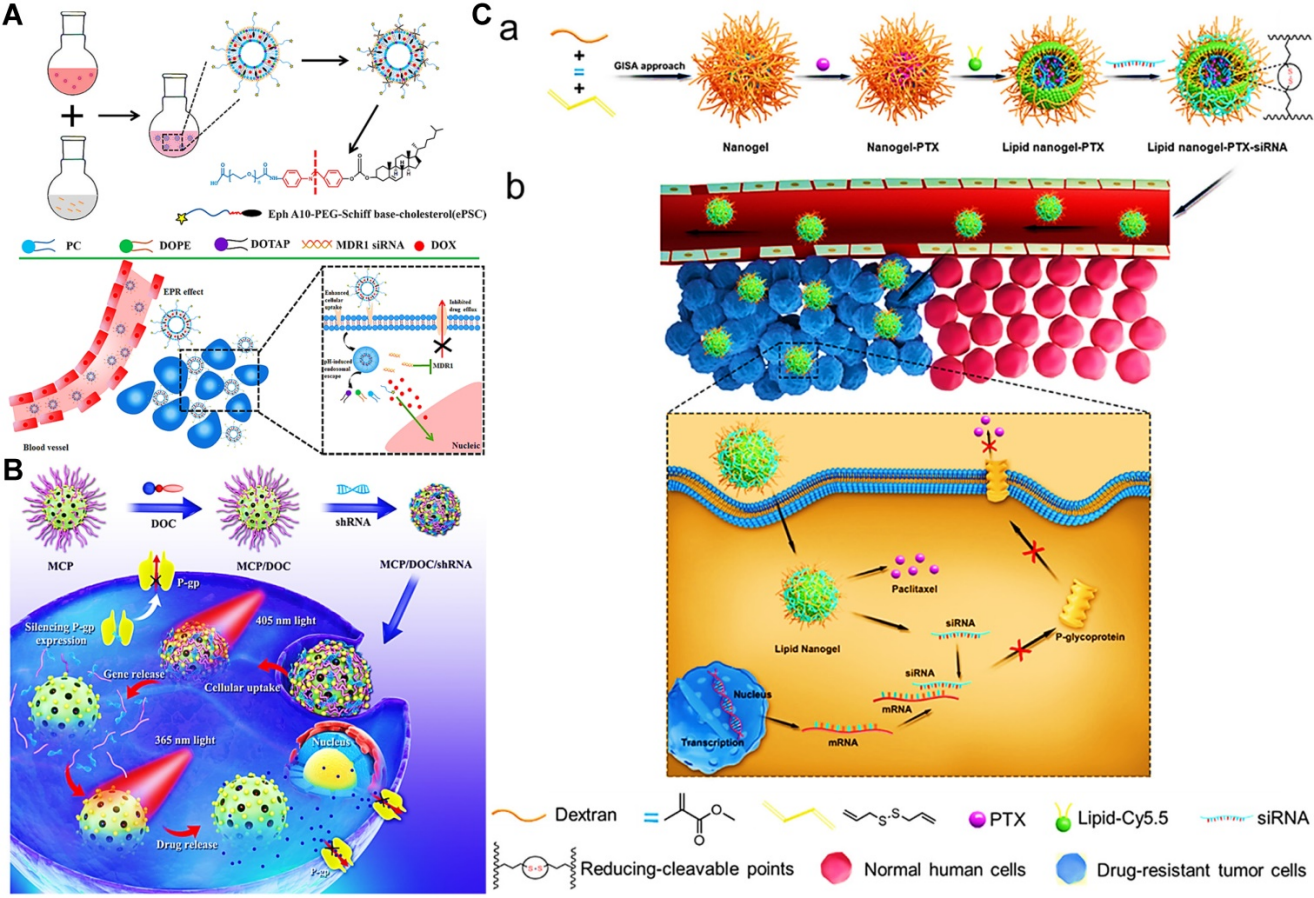
(A) Schematic illustration of the approach to overcome MDR by multifunctional liposomes co-delivering DOX and MDR1 siRNA. Reproduced with permission from Ref. 139, Copyright © 2018, American Chemical Society. (B) Schematic illustration of photo-responsive MSNs loading with shRNA and DOX for optimizing the synergistic therapy in MDR cancer cells. Reproduced with permission from Ref. 140, Copyright © 2018, American Chemical Society. (C) Schematic illustrations of a) the fabrication of MDR1 siRNA/PTX co-delivered lipid nanogels, and b) the reversal mechanism of MDR by co-delivered lipid nanogels. Reproduced with permission from Ref. 115, Copyright © 2020, Elsevier. Abbreviations: MDR1: multidrug resistance gene 1; shRNA: short-hairpin RNA; GISA: graft copolymerization-induced self-assembly; PTX: paclitaxel; siRNA: small interfering RNA.

RNA interference technology like siRNA may also be used to combine with chemotherapeutic drugs, due to its precise downregulation of the expression of MDR-related genes 135. Nevertheless, their therapeutic applications have been impeded by the short half-life in blood, lack of cellular targeting, and poor membrane permeability 136. To address these issues mentioned, different classes of nanocarriers have been constructed to concurrently delivery siRNA and chemotherapeutic drugs *in vitro* and *in vivo*, followed by the enhanced transfection efficacy of siRNA and knockout effect of MDR-related genes 99, 137, 138. For example, Zhang et al. designed actively targeted and pH-sensitive liposomes to co-load DOX and MDR1 siRNA for overcoming MDR of breast cancer (**Figure 7A**) 139. Such co-delivery liposomes increased the sensitivity of MCF-7/ADR cells to DOX via MDR1 siRNA-induced P-gp downregulation, and exhibited an enhanced therapeutic potency. In another study, to ensure prerelease of RNA molecules than chemotherapeutic drugs with a sufficient interval, Wu et al. fabricated a photo-responsive MSN loading with DOX and P-gp shRNA to overcome MDR of HepG2 liver cancer cells, by which sequential release of shRNA and DOX could be achieved by using 405 and 365 nm light irradiations, respectively (**Figure 7B**) 140. Eventually, these MSNs could result in enhanced DOX retention inside MDR cancer cells by the initial release of shRNA silencing P-gp expression, and bring out an optimized chemotherapeutic effect. Furthermore, Wang et al. designed a biomimetic lipid/DEX hybrid nanogel to co-deliver MDR1 siRNA and PTX for inhibiting PTX-resistant ovarian cancer (**Figure 7C**) 115, in which MDR1 siRNA could knock down MDR1 to promote the accumulation of PTX in cancer cells, thereby achieving an efficient inhibitory effect against highly PTX-resistant cancer cells.

#### Chemotherapeutic drugs and chemosensitizers

**Figure 8:**
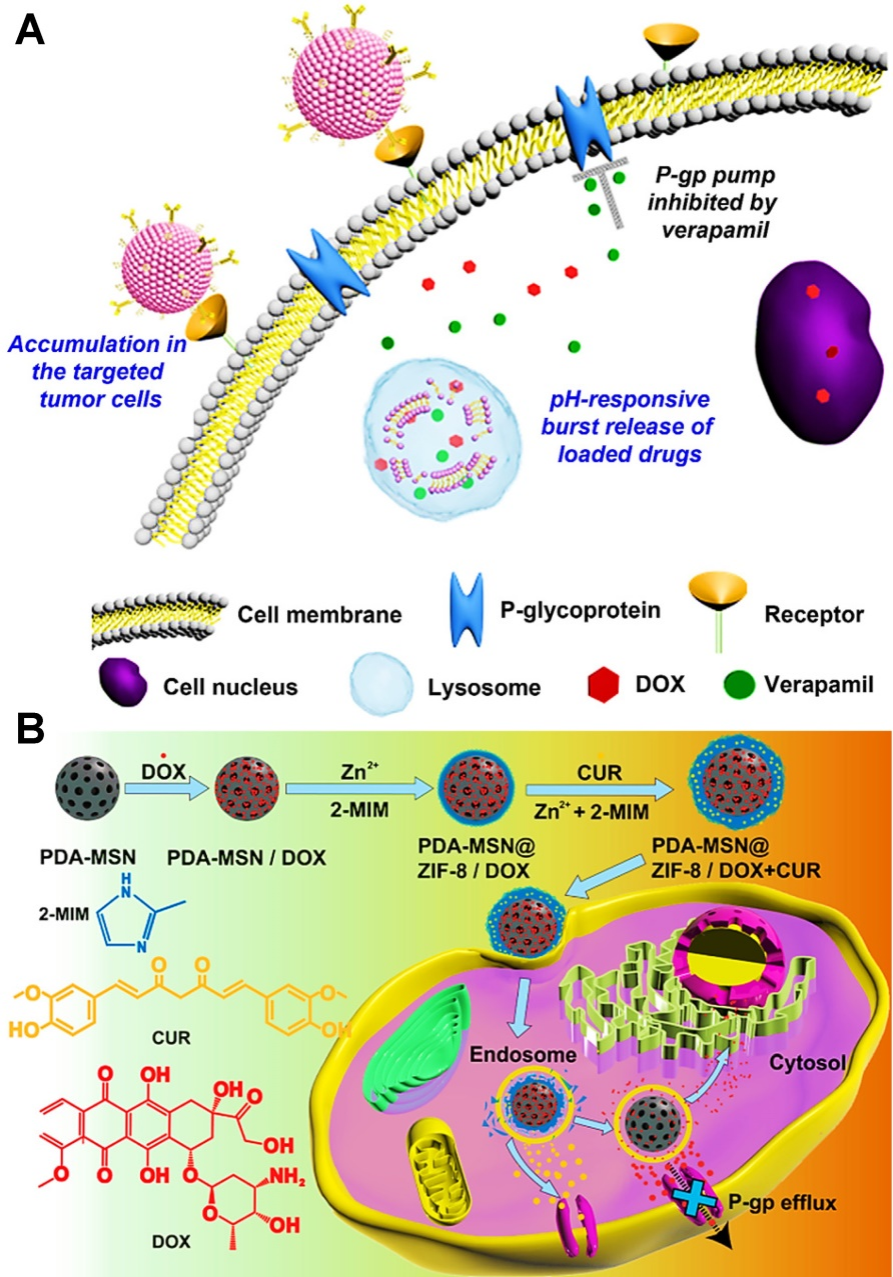
(A) Schematic illustration of the mechanism of reversal MDR by pH-responsive mixed liposomes co-delivering DOX and VER. Reproduced with permission from Ref. 110, Copyright © 2014, Elsevier. (B) Schematic diagram showing the preparation of DOX and CUR loaded hybrid nanocarriers and the synergistic MDR reversal therapy. Reproduced with permission from Ref. 145, Copyright © 2018, American Chemical Society. Abbreviations: VER: verapamil; PDA: polydopamine; MSN: mesoporous silica nanoparticle; CUR: curcumin; ZIF-8: zeolite imidazolate frameworks-8.

Therapeutic nanoplatforms containing a combination of chemical drug and chemosensitizer can be further used to realize the suppression effect of cancer MDR. Among most chemosensitizers, verapamil (VER) has been identified as a common P-gp inhibitor to reverse MDR of cancer. For instance, Liu et al. constructed pH-responsive mixed liposomes for co-delivery of DOX and VER to suppress drug resistance in breast cancer, in which the increased intracellular accumulation of DOX was attributed to the inhibition of P-gp pump function by VER (**Figure 8A**) 110; Qin et al. developed a hydrogel nanoparticle for co-encapsulating DOX and VER to significantly improve the uptake and cytotoxicity of DOX by reversing MDR in resistant tumor cells 141; Maiti et al. developed redox-responsive and core-cross-linked micelles for simultaneous delivery of DOX and VER toward drug-resistant MDA-MB-231 breast cancer cells 142, which significantly improved chemosensitivity by VER and enhanced anticancer efficacy* in vitro*. Besides, Soma et al. have reported that co-delivery of chemosensitizer cyclosporin A (CyA) and chemotherapeutic DOX with a single nanocarrier display nearly 2-fold increase of cytotoxicity, resulting from the reversal action of CyA against MDR in DOX-resistant leukemia cells 105. Some studies have also indicated that CUR served as an ideal chemosensitizer possesses the ability to reverse MDR of cancer cells through the inhibition of P-gp pump 143, 144. In a research by Wang et al., hybrid nanocarriers based on polydopamines and MSNs were prepared to simultaneously load with DOX and CUR for overcoming MDR of breast cancer (**Figure 8B**) 145. As expected, the released CUR suppressed the drug efflux function of P-gp, subsequently facilitating intracellular accumulation of DOX and synergistic effects on killing MDR tumor cells. Furthermore, disulfiram (DSF) is another type of chemosensitizer, which can be used as a P-gp inhibitor. Duan and co-workers have investigated a micelle-mediated co-delivery of DOX and DSF 81. The DSF released from this nanosystem directly decreased the P-gp activity, thus reversing MDR to sufficiently exert therapeutic effect of DOX in DOX-resistant breast cancer cells.

#### Chemotherapeutic drugs and gas therapy agents

**Figure 9:**
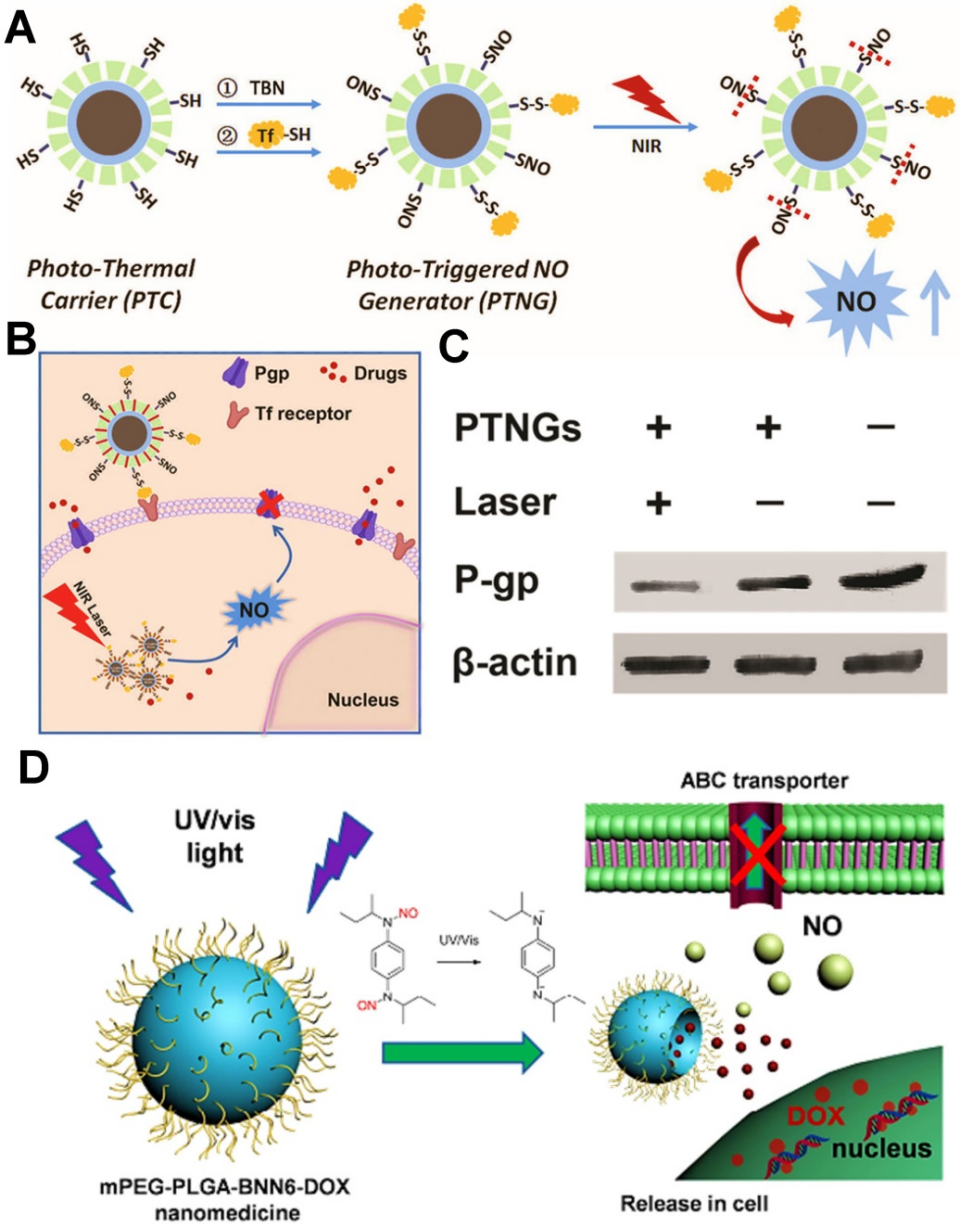
(A) Schematic illustration of the fabrication of phototriggered NO nanogenerators (PTNGs). (B) Schematic diagram of the endocytosis of PTNGs and the subsequent P-gp reversal by phototriggered release of NO. (C) Western blot for the detection of P-gp expression. Reproduced with permission from Ref. 146, Copyright © 2017, Wiley-VCH Verlag GmbH & Co. KGaA, Weinheim. (D) mPEG-PLGA-BNN6-DOX nanoparticles release NO in response to UV/Vis irradiation, which further increase the sensitivity of cancer cells to DOX via inhibiting P-gp-mediated MDR. Reproduced with permission from Ref. 108, Copyright © 2016, American Chemical Society. Abbreviations: NO: nitric oxide; Tf: transferrin; UV/vis: ultraviolet/visible.

Some gaseous therapeutic agents (e.g., NO, H_2_, SO_2_) have been served as ABC transporter inhibitors to reverse tumor MDR. Among them, NO is the most prominent gasotransmitter, playing a key role in inhibiting P-gp expression. From this, several relevant studies have reported that light-triggered NO-generating nanoplatforms can show great potential for conquering MDR cancer. For instance, Guo et al. designed intelligent near-infrared (NIR) laser-triggered NO nanogenerators for reversing DOX-resistant breast cancer (**Figure 9A-C**) 146, which were fabricated by integrating photothermal agents and S-nitrosothiols (SNO, a heat-sensitive NO donor) into a single nanoparticle. Such nanogenerators could be converted into ample heat to trigger NO release from SNO through NIR laser, and then the NO molecules further effectively reversed MDR by decreasing the expression of P-gp transporter. Consequently, the intracellular accumulation of DOX was effectively increased in MDR cancer cells. In another research by Fan et al., they proposed a NO-promoted chemosensitization of DOX strategy, which were based on a biodegradable mPEG-PLGA nanoparticles co-delivering DOX and BNN6 (an ultraviolet-visible (UV/Vis)-sensitive NO donor) for overcoming MDR in ovarian cancer (**Figure 9D**) 108. Once exposure to UV/Vis illumination, the BNN6 from these nanomedicines could decompose into NO gas to inhibit the P-gp. The generated NO further broke the nanoparticle shell to release DOX, leading to a remarkable gas/chemotherapeutic effect on defeating the MDR cancer. In addition to light triggering NO production, Wan et al. investigated a NO synthetase-mediated NO nanogenerator based on the heparin/FA nanoparticles loading with L-arginine (a NO donor) and DOX 147; Chen et al. developed a pH-sensitive NO generation liposomal system for delivering DETA NONOate (a NO donor) and PTX 112; Chu et al. constructed a GSH-triggered NO-generating polyprodrug nanoparticle by integrating CDDP prodrug and NO prodrug monomer (StNO) 148. Overall, these multifunctional nanoplatforms with NO release could enable effective reversal of MDR in cancer chemotherapy.

**Figure 10:**
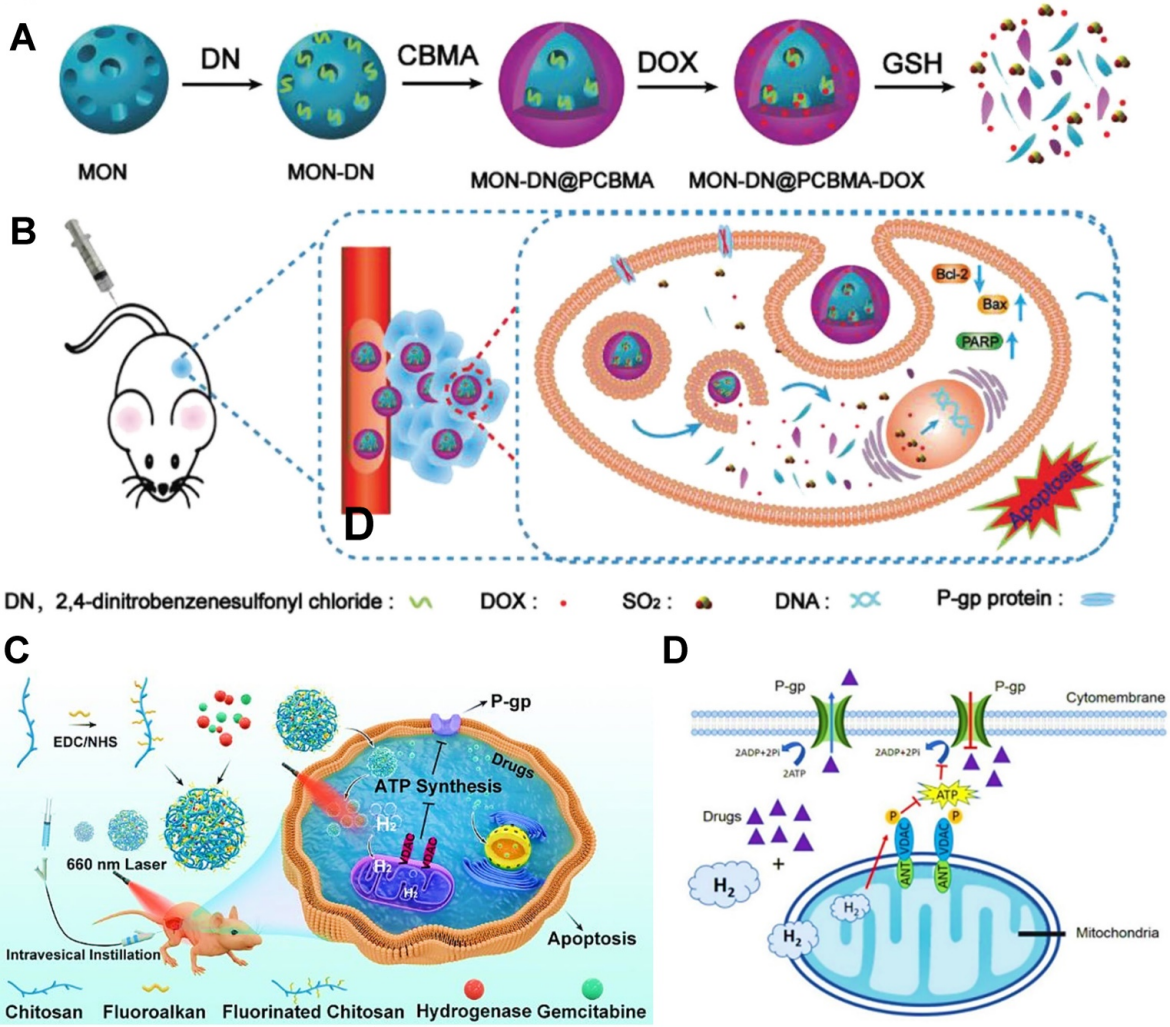
(A) Schematic illustration of the redox-responsive SO_2_-releasing nanosystem for producing synergistic effect of chemotherapy and gas therapy. (B) Mechanism of the reversal of MDR by the released SO_2_ and combination therapy. Reproduced with permission from Ref. 149, Copyright © 2020, Wiley-VCH Verlag GmbH & Co. KGaA, Weinheim. (C) Schematic illustration of the *in situ* photo-activated H_2_ nanogenerator ([FeFe]TPP/GEM/FCS) for enhanced chemotherapy of bladder cancer. (D) Mechanism of the recovery of chemosensitivity by H_2_ suppressing P-gp pump. Reproduced with permission from Ref. 107, Copyright © 2020, American Chemical Society. Abbreviations: MON: mesoporous organosilica nanoparticle; PCBMA: poly(carboxybetaine methacrylate); GEM: gemcitabine; FCS: fluorinated chitosan.

Moreover, SO_2_ or H_2_ can be used to reverse P-gp-mediated MDR in cancers. For example, Yao et al. developed a GSH-responsive SO_2_-releasing nanosystem based on mesoporous organosilica nanoparticles (MONs) and poly(carboxybetaine methacrylate) (PCBMA) (**Figure 10A**) 149, which concurrently loaded SO_2_ prodrug molecules (DN, 2,4-dinitrobenzenesulfonylchloride) and DOX. The release of SO_2_ from DN under intracellular GSH could downregulate the expression of P-gp, thus sensitizing breast cancer cells to chemotherapy (**Figure 10B**). Sun et al. proposed a photo-activated H_2_ nanogenerator based on the fluorinated chitosan 107, which co-delivered [FeFe]TPP (a catalyst of H_2_ production) and gemcitabine (GEM) for enhanced chemotherapy in bladder cancer (**Figure 10C**). Upon a 660 nm illumination, these nanogenerators could efficiently generate H_2_ to cause the reduction of efflux pump function, which attenuated drug transport capacity of P-gp and improved the therapeutic efficacy of GEM (**Figure 10D**).

### Co-delivery of chemotherapeutic drugs and pro-apoptotic agents

One of the aforementioned MDR mechanisms is closely relevant to the regulation of apoptosis or survival pathways. Generally, the repair of dysfunctional apoptotic signaling by pro-apoptotic agents is capable of bypassing MDR of cancer cells 150. Therefore, we may exploit various therapeutic nanocarriers to co-encapsulate chemotherapy drugs with apoptosis-induced compounds, thereby recovering the chemosensitivity in MDR cancer cells by influencing this classical death pathway. Ceramide (CER) as a type of apoptosis inducer plays a vital role in programmed cell death pathway. Whereas resistant tumor cells often restrain the apoptosis initiation by overexpressing glucosylceramide synthase that inactivates the CER. To address it, Van et al. used polymeric micelles to co-deliver a combination of PTX with CER against PTX-resistant ovarian cancer. Owning to CER-mediated aggravation of cell apoptosis, these co-delivery micelles could promote the PTX sensitivity in MDR cells and generate a 100-fold increase in cytotoxity 104. Likewise, van Vlerken et al. adopted PTX and CER combination therapy based on a polymer-blend nanoplatform for both resistant ovarian and breast cancers, which successfully circumvented MDR by CER-mediated an enhancement in apoptotic signaling 150. Additionally, since the hyper-expression of NF-κB may cause drug resistance of cancer cells, some pro-apoptotic agents related to NF-κB signaling pathway have been developed to block chemotherapeutic resistance in cancer treatment 151. A previous study has indicated that, co-delivery of DOX and pyrrolidine dithiocarbamate (PDTC, a NF-κB inhibitor) using chitosan-based nanoparticles can successfully reverse MDR of human liver cancer cells by PDTC-induced inactivation of NF-κB pathway 152. Cheng et al. also reported co-delivery of DOX and PDTC by a pH-sensitive polymeric nanoparticle based on poly(ortho ester urethanes) copolymers for overcoming MDR in breast cancer 153. The acid-triggered release of PDTC could significantly reverse MDR and increase intracellular DOX accumulation through inhibiting NF-κB pathway, thus resulting in enhanced DOX-induced cytotoxicity and apoptosis in resistant breast cancer cells.

### Co-delivery of chemotherapeutic drugs and peptides/proteins

**Figure 11:**
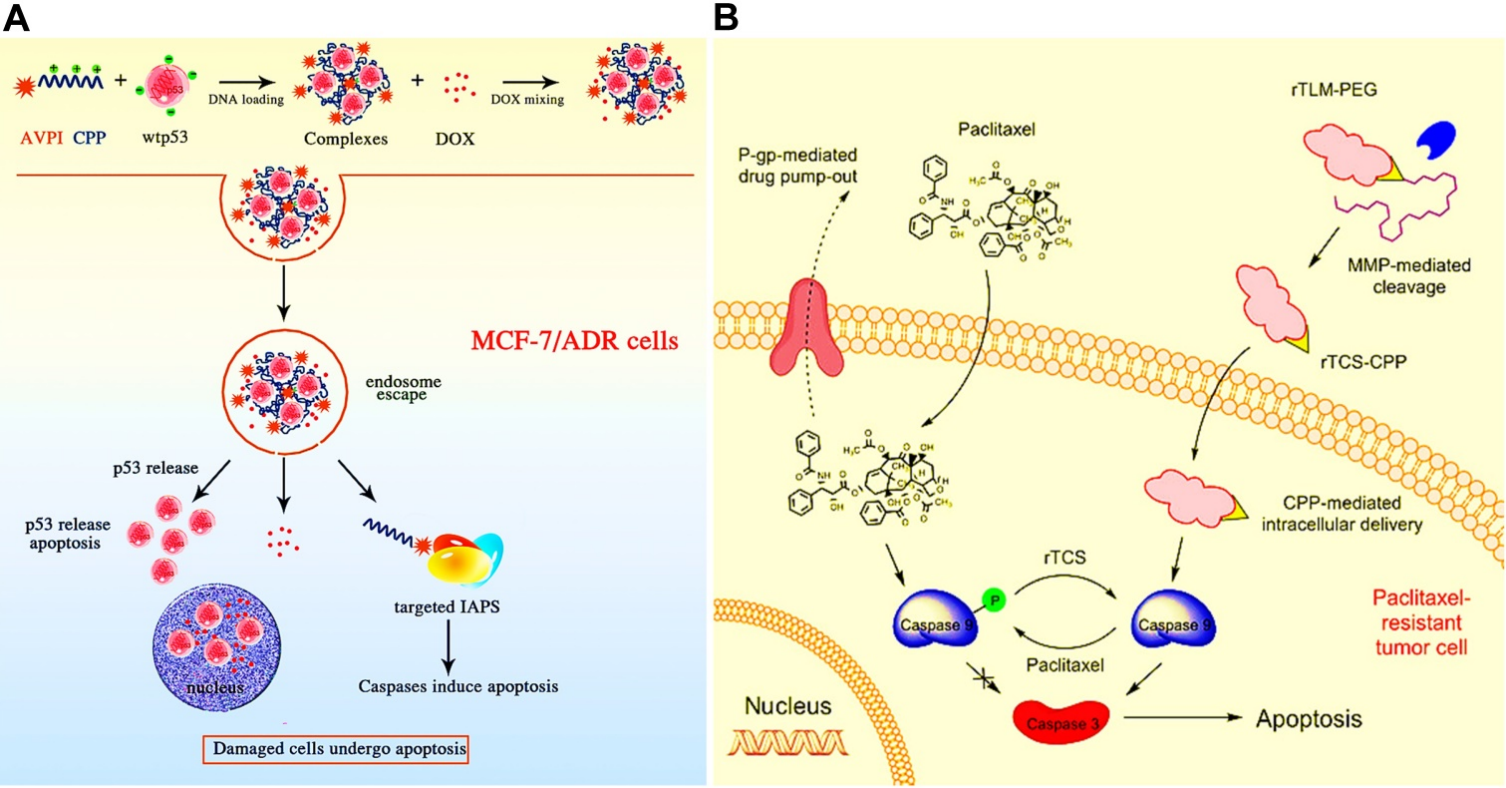
(A) Schematic illustration of co-delivery of cell-penetrating AVPI-R8/p53 DNA nanocomplex for overcoming drug resistance. Upon nanocomplex entered the resistant MCF-7/ADR cells, the p53 DNA and AVPIR8 chimeric peptide released from endosomal escape could induce caspase-dependent apoptosis, thereby easing DOX-resistance to potentiate the toxicity of resistant tumor cells. Reproduced with permission from Ref. 155, Copyright © 2014, American Chemical Society. (B) Schematic illustration of co-delivery of TCS (a ribosome-inactivating protein) and PTX by a liposomal carrier for reversal of chemoresistance in lung cancer. Reproduced with permission from Ref. 111, Copyright © 2017, American Chemical Society. Abbreviations: AVPI: the Smac N-terminal tetrapeptide; CPP: cell-penetrating peptide; IAPs: inhibitors of apoptosis proteins; TCS: trichosanthin; MMP: matrix metalloproteinase.

Peptides or proteins with high selectivity and potency are also identified as a model drug that defended the MDR mechanisms 154. Many co-delivery nanocarriers have been designed to co-encapsulate peptides/proteins and chemotherapeutic agents against the tough MDR tumor. For example, Haggag et al. constructed liposomes co-delivering DOX and a Ras-related nuclear protein (Ran-GTP) inhibitory peptide for inhibiting DOX-resistant breast cancer 41. In this study, the aberrant repair of DNA damage by Ran-GTP resulted in the occurrence of drug-resistance mechanism. Herein, the use of a Ran-GTP inhibitory peptide could effectively reverse the Ran-overexpression mediated MDR via inhibiting the DNA repair function of Ran, thus achieving enhanced DOX sensitivity and improved antitumor effect. Moreover, some chimeric peptides can also be served as a potent pro-apoptotic agent for overcoming tumor MDR. In a work, co-delivery of AVPIR8 chimeric peptide and p53 DNA using the self-assembly nanoparticles significantly increased the DOX sensitivity in the resistant MCF-7/ADR cells (**Figure 11A**) 155. In addition to peptides, several therapeutic proteins (e.g., trichosanthin (TCS)) are emerging as candidates for overcoming cancer MDR 156. For instance, a co-delivery liposomal carrier carrying a combination of TCS and PTX were constructed to circumvent PTX resistance in A549 lung cancer cells by reversing PTX-caused caspase 9 phosphorylation and inducing caspase 3-dependent apoptosis, and successfully suppressed the PTX-resistant A549 tumor growth* in vivo* (**Figure 11B**) 111.

## Conclusion and outlook

With the accelerated advance in personalized and precision medicine, the exploitation of nanotechnology-based drug delivery systems for cancer therapy is also becoming a booming research field. At present, a variety of nanoscale transport vehicles with different functional characteristics have been born at the right moment, which endue these drug delivery platforms with tumor selective targeting, tumor environmental or external physical stimulus responsiveness. Such an intelligent controlled-release system can better target tumor cells and be further internalized to release drugs in response to specific tumor physiological conditions or external physical stimuli. In addition, while designing these nanomedicines, we may consider either directly wrapping therapeutic drugs into the carrier under physical action or doping the drug molecules into the carrier by chemical graft. Here, MDR as a common feature of many cancers has undoubtedly become one of the most intractable challenges on the road to cancer treatment, which seriously affects the anticancer effect of chemotherapy, resulting in a high recurrence rate and a low survival rate in clinical cancer cases. Therefore, two drug models with different pharmaceutical and functional properties are considered to be integrated into an intelligent nanoplatform to combat tumor MDR. With the advantages of targeting and stimulus response of the nanoplatforms, therapeutic cargoes will be expected to be transported into drug-resistant tumor cells, and the synergistic effect of dual drugs can successfully reverse MDR and produce enhanced anti-tumor effects.

Nonetheless, there are still some severe challenges in the research on co-delivery of dual-drug using multifunctional nanoplatforms for treating multidrug resistant cancers. First, although nanomaterials can help drugs penetrate and accumulate within the resistant tumors, the actual number of drugs that can be delivered into tumor tissues is still limited. So, we also need to redouble our efforts in the development of drug delivery systems for combination therapy. Second, it is crucial to select the optimal combinatorial ratio of dual drugs, which directly affects the inhibitory effects on tumor cells, including synergistic, antagonistic, and non-additive effects. Moreover, due to the influence of complex *in vivo* physiological barriers, it is very likely to cause the changes of combinatorial ratio at any time, thus affecting the therapeutic effect of tumor. Third, the sequence and timing of each drug release from nanovehicles is also worth exploring, which directly affects how individual drug works. For instance, when using a nanocarrier co-delivering siRNA and DOX to treat multi-drug resistant cancers, it can be planned to first release siRNA to reverse MDR by inhibiting P-gp level, so as to increase the sensitivity and retention of subsequent release of DOX, and to maximize the anticancer toxicity of DOX. In addition, it is also critical to select the type of individual therapeutic agent in drug delivery systems. When two chemotherapeutic drugs are used in combination, the cross-tolerance generated in combination therapy should be avoided, instead of the dual-drug combination group that can generate collateral sensitivity. Last, when nanocarriers unload the therapeutic cargoes inside the tumor tissues or cells, these free-state drugs will still be affected by MDR mechanisms, likely resulting in drug molecules being inactivated or pumped out of cells in large quantities. Thus we may select appropriate MDR reversal agents that alleviate chemotherapy tolerance, such as surfactants, small molecule drugs, nucleic acid drugs, gaseous therapeutic agents or peptides/proteins, to restore the sensitivity of drug-resistant tumor cells to chemotherapeutic drugs.

Besides, taking nanotechnology-based combination therapeutics from bench to bedside is extremely challenging nowadays. The main reason for this is that, the development of co-delivery nanomedicines usually holds significantly higher level of complexity and difficulty in manufacturing, characterization and assessment, which will incur high costs and low benefits for future large-scale production in industry. For example, the precise ratio control of dual drugs in nanosystems is a big challenge in production, often resulting in batch-to-batch inconsistency. Moreover, it has been found that some certain nanomaterials are relevant to oxidative stress, undesirable inflammatory responses, and genotoxicity 18. Accordingly, the latent adverse effects in nanomaterials should be also considered while designing combination therapies. Based on these issues, the clinical application of combinatorial nanomedicines still remains immature and unavailable, which makes it urgent to do a lot of pre-clinical basic work to increase the possibility of co-delivery nanomedicines to clinical translation in the future.

Nevertheless, several nanoplatforms possess the potential to clinically develop co-delivery nanomedicines. As is well-known, the first anticancer nano-drug approved by the U.S. Food and Drug Administration (FDA) was liposome-based Doxil, and liposomes are currently widely used as nanocarriers for the clinical development of anticancer nanomedicines. The reasons why liposomes are favored in clinical practice may be as follows: (1) from the perspective of bionics, the phospholipid bilayer of liposomes is similar to the structure and components of animal cell membranes, which is more conducive to the delivery of drugs under complex physiological environment; (2) the synthesis of liposomes is controllable and regenerative, and its clinical evaluation and monitoring system has been well established; (3) the rich experience in the production and management of liposome drugs can be further used for reference. Therefore, we believe that liposome-based co-delivery nanomedicines that can eliminate cancer MDR will be available on the market in the near future. Furthermore, other FDA-approved carrier materials, such as PLGA and Fe_3_O_4_, also have unique advantages in developing co-delivery nanomedicines. In conclusion, with the rapid development of nanotechnology, more and more nanomaterials will be applied to clinical research, which makes it more possible to adopt co-delivery nanomedicines for the clinical treatment of multidrug resistant cancers.

## Figures and Tables

**Table 1:** Various nanoformulations for co-delivery of dual anticancer therapeutics

Nanoformulation	Combined therapeutics	Drug-loading mechanism	Target	Refs.
Polymeric micelle	THZ/DOX	Hydrophobic forces	Breast cancer	34
	DOX/PTX	Electrostatic forces/hydrophobic forces	Lung cancer	35
	PTX/TR3 siRNA	Hydrophobic forces/electrostatic forces	Pancreatic cancer	36
	Msurvivin T34A gene/DOX	Electrostatic forces/hydrophobic forces	Melanoma	37
Polymeric nanoparticle	Retinoic acid/DOX	Hydrophobic forces	Breast cancer	38
	AntimiR10b/antimiR-21	Electrostatic forces	Breast cancer	39
	CDDP/RAPA	Hydrophobic forces	Melanoma	29
	MiR-34a/DOX	Electrostatic forces/hydrophobic forces	Breast cancer	40
Liposome	RanGTP/DOX	Passive diffusion	Breast cancer	41
	PTX/TRAIL	Hydrophobic forces/electrostatic absorption	Melanoma	42
	VEGF siRNA/PTX	Electrostatic absorption/hydrophobic forces	Breast cancer	43
	DFO/YC-1	Passive diffusion/hydrophobic forces	Pancreatic cancer	44
MSN	CPT/DOX	Hydrophobic forces/covalent binding	Cervical cancer	45
	P-gp siRNA/DOX	Electrostatic absorption/hydrophobic forces	Breast cancer	46
Fe_3_O_4_ nanoparticle	DOX/CDDP	Covalent binding	Breast cancer	47
Nanogel	CDDP/DOX	Chelation/electrostatic interaction/π-π stacking interaction	Breast cancer	48
	Epigallocatechin gallate/siRNA	Electrostatic interaction	Breast cancer	32
NGO	ADM/anti-miR-21	Hydrophobic forces/electrostatic forces	Breast cancer	49

**Table 2:** Various dual-drug combinations in nanocarriers for reversing cancer MDR

Nanoformulation	Drug combinations	Reversal mechanism of MDR	Target	Refs.
Polymeric micelle	DOX and HCPT	Evade the recognition of drug efflux pumps by π-π stacking and collateral sensitivity between dual drugs	Breast cancer	27
	DOX and APA	Recover the chemosensitivity by competitively inhibiting P-gp activity	Breast cancer	55
	DOX and LPA	Inhibit MDR transporters by LPA interacting with the substrate-binding site	Breast cancer	103
	DOX and TPGS2000	TPGS2000-mediated inhibition of P-gp pump activity by reducing MMP and depletion of ATP	Breast cancer	77
	MDR-1 siRNA and DOX	Bypass P-gp-mediated DOX resistance through siRNA silencing P-gp	Breast cancer	76
	DOX and DSF	Evade drug resistance by disulfiram blocking the activity of P-gp	Breast cancer	81
	Ceramide and PTX	Overcome PTX resistance by ceramide-mediated aggravation of cell apoptosis	Ovarian cancer	104
Polymeric nanoparticle	DOX and CyA	Inhibit MDR by CyA directly binding to P-gp drug pump	Leukemia	105
	DOX and CUR	Reverse MDR by the downregulated expression of P-gp	Ovarian cancer	98
	PDTC and DOX	Block chemoresistance by inhibiting NF-κB signaling pathway	Liver cancer	106
	GEM and [FeFe]TPP	Reverse MDR by H_2_ causing the reduction of P-gp efflux pump function	Bladder cancer	107
	DOX and BNN6	Overcome DOX resistance by NO inhibiting the expression of P-gp	Ovarian cancer	108
Liposome	CDDP and Bcl-2/Survivin/P-gp siRNAs	Reverse MDR by blocking apoptosis and P-gp mediated resistance pathways	Ovarian cancer	109
	DOX and VER	Overcome DOX resistance by VER inhibiting P-gp activity	Breast cancer	110
	RanGTP and DOX	Reverse Ran-mediated MDR by inhibiting the Ran DNA damage repair function	Breast cancer	41
	PTX and TCS	Overcome PTX resistance by TCS reversing PTX-caused caspase 9 phosphorylation and inducing caspase 3-dependent apoptosis	Lung cancer	111
	PTX and DETA NONOate	Reverse PTX resistance by NO-mediated downregulation of P-gp	Lung cancer	112
MSN	DOX and CTAB	CTAB-mediated inhibition of P-gp activity by depletion of ATP	Breast cancer	113
	P-gp siRNA and DOX	Recover DOX sensitivity by siRNA silencing the expression of P-gp	Breast cancer	114
Nanogel	CDDP and DOX	Overcome drug resistance by synergistic chemotherapy	Breast cancer	48
	PTX and MDR1 siRNA	Recover PTX sensitivity by siRNA knocking down MDR1	Ovarian cancer	115

## Abbreviations

MDR: multidrug resistance
P-gp: P-glycoprotein
EPR: enhanced permeability and retention
MSNs: mesoporous silica nanoparticles
THZ: thioridazine
DFO: deferoxamine
YC-1: lificiguat
DOCA: deoxycholate
PLL: poly(L-lysine)
ADM: adriamycin
PTX: paclitaxel
PDPA: poly(2-(diisopropyl amino)ethyl methacrylate)
PEG: poly(ethylene glycol)
DOX: doxorubicin
PLGA: poly(lactic-co-glycolic acid)
PLA: poly-L-lactide
RAPA: rapamycin
CDDP: cisplatin
CS: chondroitin sulfate
DEX: dextran
HA: hyaluronic acid
PpIX: protoporphyrin IX
APA: Apatinib
CPT: camptothecin
DAT: dexamethasone
DTX: docetaxel
DSPE-PEG2000: 1,2-Distearoyl-sn-glycero-3-phosphoethanolamine-N-methoxy(polyethyleneglycol)
CHOL: cholesterol
TRAIL: tumor necrosis factor-related apoptosis-inducing ligand
Fe_3_O_4_: iron oxide
CUR: curcumin
NGO: graphene oxide
FA: folate
TF: transferrin
TFR: transferrin receptor
GSH: glutathione
ATP: adenosine triphosphate
ABC: ATP-binding cassette
HCPT: 10-hydroxycamptothecin
TKIs: tyrosine kinase inhibitors
LPA: lapatinib
TPGS: D-α-tocopheryl polyethylene glycol 1000 succinate
NO: nitric oxide
H_2_: hydrogen
SO_2_: sulfur dioxide
MMP: mitochondrial membrane potential
CTAB: cetyl trimethyl ammonium bromide
VER: verapamil
CyA: cyclosporin A
DSF: disulfiram
NIR: near-infrared
SNO: S-nitrosothiols
UV/Vis: ultraviolet-visible
GEM: gemcitabine
CER: ceramide
PDTC: pyrrolidine dithiocarbamate
Ran-GTP: Ras-related nuclear protein
TCS: trichosanthin
FDA: Food and Drug Administration
